# Everybody's Page

**Published:** 1889-10-19

**Authors:** 


### Everybody's Page.

A third of the deaths in the French army are due to typhoid fever.

In horses the pulse beats forty times a minute when the animal is at rest; in an ox from fifty to fifty-five times; and in sheep and pigs about seventy to eighty times a minute.

Antimony is one of the oldest minerals known. Recent discoveries in Assyria show that this metal was used in very ancient times in the manufacture of vessels and ornaments. Many things which were generally supposed to be tin are now proved to be formed of antimony.

A large quantity of what is called lime-juice is derived neither from limes nor lemons, but from the bergamot, another variety of the *citrus* family. Montserrat, in the West Indies, is one of the few places where limes are cultivated on a large scale for the production of lime-juice.

Copal is a gum-resin obtained from certain tropical and sub-tropical trees. In some cases it is obtained from living or decaying trees, but the largest supply is obtained in a semi-fossil state in districts where there are no trees now, but which at one time were occupied by large forests.

Till recently ozokerite, or mineral wax, was not known to exist anywhere out of Moldavia and Galicia. Three years ago, however, a deposit was discovered in the territory of Utah, about 114 miles east of Salt Lake City. The mine covers at least 150 acres, and about 1,000 tons can be taken from it in a year.

When sponges are found and have been torn from their bed, they are spread about the deck of the ship that has gone to investigate the sponge grounds, so that as they die and decompose the gelatinous substance of which the animal is formed may run off freely. After a few days they are taken to the shore and deposited in small pens of water for further cleansing.

Saccharin deceives the human palate, but not that of cats, dogs, and wasps. Liebreich has demonstrated that bees and dogs can distinguish unerringly between food containing saccharin and that which is free from it. Some cats also declined to touch their food when it was sweetened with saccharin; when a new supply, free from the obnoxious sweetening, was substituted, they smelled it carefully, but finally ate it.

Balls of closely matted hair are sometimes taken from the stomach of horses and cows and other animals that lick their young; but it is new to find any human being swallowing hair. A case, however, is recorded of a German schoolgirl, who bit the ends off her hair and swallowed them under the impression that it would make her voice clearer. Some of her companions did the same, but they were more fortunate than this girl, in whose stomach a hair tumour formed, which had to be removed—a very disagreeable proceeding.

The late Mattieu Williams records a simple cure for burns, even though they are of a severe character. A workman engaged in the distillation of cannel coal got badly burned about the face in an explosion. The foreman dragged him to a tank of crude oil that was near, and smeared his head and face with the greasy, tarry mixture. The application was frequently renewed, and the patient suffered very little pain, except when the oiling process was delayed. In a few days he was able to return to work, and though his skin peeled off as if he had suffered from scarlet fever, no permanent injury ensued. This crude oil may be imitated in a more agreeable form by mixing a little carbolic acid with vaseline.

It is said that the Caucasian petroleum wells are gradually becoming exhausted.

Soapstone is one of the most valuable minerals known. It is used for dressing skins, leather, and gloves; for making slate pencils, ovens, and the linings of limekilns; and is also employed in the manufacture of soap, paper, and india-rubber.

"Cognac" derives its name from an ancient town in the Champagne district of France. The phylloxera has, however, ruined the industry, and of the brandy in the market which bears the name of Cognac hardly any comes from the original district.

It is fifteen years since the Trappist monks began to plant eucalyptus trees in the Roman Campagna. Now several hundred acres are covered with them, and, as a result, there has been a large decrease in the malarial fevers once so common in the district.

A French writer holds that saccharin has a distinct value as an antiseptic. If added to solutions in the proportion of one to five hundred parts, it prevents the formation of low organisms. A dentifrice may be easily made by dissolving six per cent. of saccharin in water, and mild solutions are useful in various ways.

A workman, employed at an American hotel, recently surprised a by-stander by warming a kettle of coffee in an original manner. He dug a hole in a pile of sand, placed a lump of lime in it, and sprinkled some water on the lime. He then put the kettle on the mixture, and banked up some sand round it. In a few minutes he announced to a companion that the coffee was a "bilin." Here was a practical chemist in a humble way.

If there is an unpleasant task in the whole range of domestic economy it is the washing of chamois skins that have become greasy and dirty, and, in fact, many people throw them away rather than take the trouble to clean them. The following method makes the task much easier: Soak the skin in water with which a fair quantity of ammonia has been mixed. After twelve hours of this bath, rinse it, and then wash it in an ample supply of water and white castile soap. When dry it will be almost as good as new.

Dr. Symes Thompson in his recent lectures on "Heredity," pointed out the route taken in the inheritance of consumption. The disease follows the sex of the first sufferer. Thus, if the mother is of a phthisical habit, the daughters are likely to inherit the tendency to phthisis in a much more pronounced form than the sons. On the other hand, where the disease comes from the father's side, the sons will very probably succumb to it, while the daughters may entirely outgrow any danger.

What are kid gloves made of? Of the skin of a kid, is of course the obvious answer; but Dr. Mark Nardyz, a Greek physician, now in Philadelphia, tells another tale. He says that our "kid" gloves are often made of human skin, and that the tanning of the human epidermis goes on largely in France and Switzerland. The skin of a child being finer and softer, is of more value than that of an adult. Human skin is indeed tougher than we are inclined to believe when we are wearing it, and the skin of a man's back makes a good sole leather. Dr. Nardyz declares that at one time tanning of human skin went on in Massachusetts, but General Ben Butler heard of it, and effectually put a stop to the industry. They might do worse than send General Ben Butler on a mission to France and Switzerland, if what the Greek doctor says be true.

